# Nonsense-mediated mRNA decay: a key regulatory system engaged in cancer

**DOI:** 10.1186/s12964-025-02503-6

**Published:** 2025-11-11

**Authors:** Hongyu Pan, Xinyu Wu, Bingshuang Hu, Huihua Xiong, Yao Luo, Jian-Kang Zhou

**Affiliations:** 1 Department of Pathology and Pathophysiology, School of Basic Medical Sciences, Chengdu Medical College, Chengdu, 610500 China; 2 School of Clinical Medicine, Chengdu Medical College, Chengdu, 610500 China; 3 Department of Oncology, Tongji Hospital, Tongji Medical College, Huazhong University of Science and Technology, Wuhan, 430030 China; 4 Department of Laboratory Medicine, Sichuan Clinical Research Center for Laboratory Medicine, West China Hospital, Sichuan University, Chengdu, 610041 China; 5 Institute of Geriatric Cardiovascular Disease, Chengdu Medical College, Chengdu, 610500 China

**Keywords:** Nonsense-Mediated mRNA decay (NMD), Cancer progression, Premature termination codon (PTC), Alternative splicing, Tumor microenvironment, Therapeutic targeting

## Abstract

Nonsense-mediated mRNA decay (NMD) is a critical cellular surveillance mechanism that prevents the translation of defective or deleterious proteins. The regulation of NMD, including both its activation and the evasion of its target mRNA, is intricately linked to tumorigenesis. When NMD becomes overactivated, it can downregulate tumor suppressor transcripts, or eliminate immunogenic peptides, thereby promoting tumor growth and immune evasion. In contrast, reduced or defective NMD can stabilize mutated oncogene transcripts and drive tumor progression. This review provides a comprehensive overview of the physiological mechanisms of NMD, its diverse substrate features, and its regulatory dynamics. We further focus on recent advances in clarifying the interplay between NMD and tumor biology. By integrating the current findings, we aim to provide an insightful understanding of how NMD contributes to tumor initiation, tumor progression, and immune modulation.

## Introduction

Understanding how cells control gene expression to prevent the production of deleterious proteins has long been a central focus of cancer biology. Even subtle errors in gene expression within eukaryotic cells can result in profound effects on cellular function and homeostasis. Hence, the accurate control of gene expression is essential for maintaining normal cellular functionality. In addition to transcriptional regulation, cells rely on dedicated RNA quality-control pathways. Nonsense-mediated mRNA decay (NMD) plays a central role through the elimination of aberrant transcripts and the preservation of proteome integrity [1].

NMD was initially discovered in yeast [2, 3], where nonsense mutations in genes such as URA3, URA1, HIS4, and LEU2 accelerated the decay of the corresponding mRNAs [3, 4]. Later work confirmed that this mechanism is conserved in mammals [5], where transcripts containing premature termination codons (PTCs) undergo selective decay [6, 7]. Mechanistically, translation termination at a PTC recruits the core NMD factor up-frameshift protein 1 (UPF1) together with eukaryotic release factors (eRF1 and eRF3), and the serine/threonine kinase suppressor with a morphogenetic effect on genitalia family member 1 (SMG1) to form the SURF complex (SMG1–UPF1–eRF1–eRF3), which phosphorylates UPF1 and triggers transcript degradation [8]. This process generally occurs during the first round of translation, when ribosomes encounter a PTC on a newly exported mRNA that often still contains exon junction complexes (EJCs) [9].

PTCs, which are frequently produced by nonsense mutations or splicing errors, disrupt normal protein synthesis and destabilize mRNA. These defects affect a substantial proportion of coding transcripts and are often corrected through NMD-mediated degradation [7]. PTC insertions can lead to reduced full-length protein expression, the generation of truncated peptides, and overall destabilization of mRNA. A substantial fraction of disease-associated mRNA transcripts is subject to NMD [6, 10]. Although initially characterized as a quality control mechanism, NMD is currently recognized as a broader regulatory pathway that contributes to transcriptome homeostasis [11]. In addition to eliminating aberrant transcripts, NMD fine-tunes gene expression by selectively degrading certain physiological mRNAs, allowing cells to respond to functional demands and maintain genetic balance. Furthermore, the combination of alternative splicing with NMD (AS-NMD) provides a widely used strategy to control transcript levels and maintain cellular homeostasis [12].

In this review, we aim to explore the molecular mechanisms underlying NMD, characterize its diverse substrates, and discuss how this pathway affects tumor biology, as well as its involvement in immune modulation, alternative splicing, and the regulation of tumor microenvironments.

### Origin and physiological mechanism of NMD

In 1993, researchers reported that human triosephosphate isomerase (TPI) mRNA levels were reduced to approximately 20% of normal levels because of frame-shifting and nonsense mutations into PTCs. These PTCs cause premature translation termination and a marked decrease in nuclear mRNA abundance [13]. The same year, studies in yeast revealed that NMD depends on the UPF1 gene, the product of which plays a central role in degrading transcripts containing PTCs [4]. UPF1 was proposed to function as part of a quality control system that prevents the accumulation of truncated or potentially toxic polypeptides [4, 14]. Subsequent research on NMD has expanded across a wide range of organisms and has explored its decay pathway, molecular mechanism, and substrate specificity in greater depth. With an improved understanding of NMD, its role in diseases, especially in cancers, has attracted increasing amounts of scientific attention [15, 16]. The regulation of NMD in disease processes and its potential therapeutic interventions are currently active areas of investigation. This section outlines the typical physiological mechanisms that govern NMD activity.

#### EJC-dependent NMD

NMD is intrinsically linked to the pioneer round of translation, wherein the cap-binding complex protein (CBP) proteins CBP80 and CBP20 coordinate initial mRNA scanning. At this stage, the surveillance machinery distinguishes premature stop codons from natural stop codons before steady-state translation begins [9, 17]. A key regulatory step in this process is the phosphorylation of UPF1, which activates the decay process [1, 18]. During translation termination, UPF1 interacts with eRF1 and eRF3 and then binds to SMG1 to forms the SURF complex [8]. SMG1, a phosphoinositide 3-kinase-associated kinase, phosphorylates UPF1, and enables the degradation of aberrant transcripts [19, 20]. NMD typically occurs post splicing [21]. Upon recognition of a PTC during translation, the SURF complex is recruited to initiate the decay process [22, 23].

The recognition of PTC-containing mRNAs is largely mediated by the EJC [24], which is deposited approximately 20–24 nucleotides upstream of exon–exon junctions during pre-mRNA splicing [21]. When a PTC is located upstream of an EJC, the transcript becomes a target for NMD. This process involves the recruitment of up-frameshift 3 homolog B (UPF3B) [25], a core EJC component that interacts with eRF3a and eRF1 to form the ternary surveillance complex [26]. The C-terminal domain of UPF3B binds directly to the EJC [27], creating a platform for downstream activation.

Up-frameshift protein 2 (UPF2) subsequently binds to UPF3B and UPF1 by engaging with the SMG1C complex (a complex containing SMG1, SMG8, and SMG9). This interaction promotes the formation of the SMG1C-UPF1-UPF2 complex, and facilitates SMG1 mediated UPF1 phosphorylation [28–30]. Once phosphorylated, UPF1 serves as a scaffold to recruit downstream effectors. It can interact with SMG6, a nucleic acid endonuclease that cleaves aberrant mRNAs near PTCs via its 14-3-3-like structural domain or interact with the SMG5-SMG7 complex [31]. The SMG5–SMG7, complex subsequently recruits the decapping complex (DCP) and the deadenylation complex (CCR4-NOT), which removes cap-binding complexes and poly(A) tails, driving exonucleolytic decay [32]. These coordinated steps ensure that transcripts carrying PTCs efficiently and selectively degraded.

#### EJC-independent NMD

Although the typical NMD pathway is closely associated with EJCs, increasing evidence has shown that NMD can also proceed in an EJC-independent manner [24]. This alternative pathway was first demonstrated when aberrant mRNAs with artificially extended 3’-untranslated regions (3’UTRs) underwent degradation despite translation terminating at a normal stop codon without a downstream EJC [33, 34]. In this process, UPF1, which is highly enriched on transcripts with unusually long 3′UTRs, plays a central role by recognizing these features and triggering their degradation via NMD [35–37].

Extended 3’UTRs are generally associated with NMD susceptibility. However, transcriptomic analyses have shown that many endogenous mRNAs with ultralong 3’UTRs are resistant to degradation [38]. These findings suggest that the 3’UTR length alone is not sufficient to trigger NMD. Instead, NMD activation may depend on the presence of multiple exon junctions within the extended 3’UTR [39]. This resistance indicates the existence of evolved mechanisms that are able to evade surveillance. For instance, these mRNAs may contain cis-acting inhibitory elements or engage in alternative translation termination, such as that involving Sup35p, thereby increasing transcript stability even in the presence of premature termination signals [40].

The precise determinants of whether a long 3’UTR activates or evades NMD remain under investigation. One proposed mechanism involves the number and position of exon–exon junctions within the extended 3’UTR [39]. In some cases, retained introns in the 3’UTR can carry EJCs that persist after translation and contribute to NMD activation [41]. However, studies in human cells have shown that the presence of a 3’UTR intron alone is neither necessary nor sufficient for activation, although it may still influence the process [39].

At the molecular level, EJC-independent NMD still depends on the same core NMD factors, particularly UPF1, and its regulation by SMG1-mediated phosphorylation [42]. In mammalian cells, both phosphorylation and subsequent dephosphorylation are essential for NMD activity [42–44]. Notably, this pathway appears to exhibit heightened sensitivity to the intracellular levels of UPF2 and UPF3b. Compared with the classical pathway, reducing either factor disproportionately impairs EJC-independent NMD [26, 45].

**Fig. 1:**
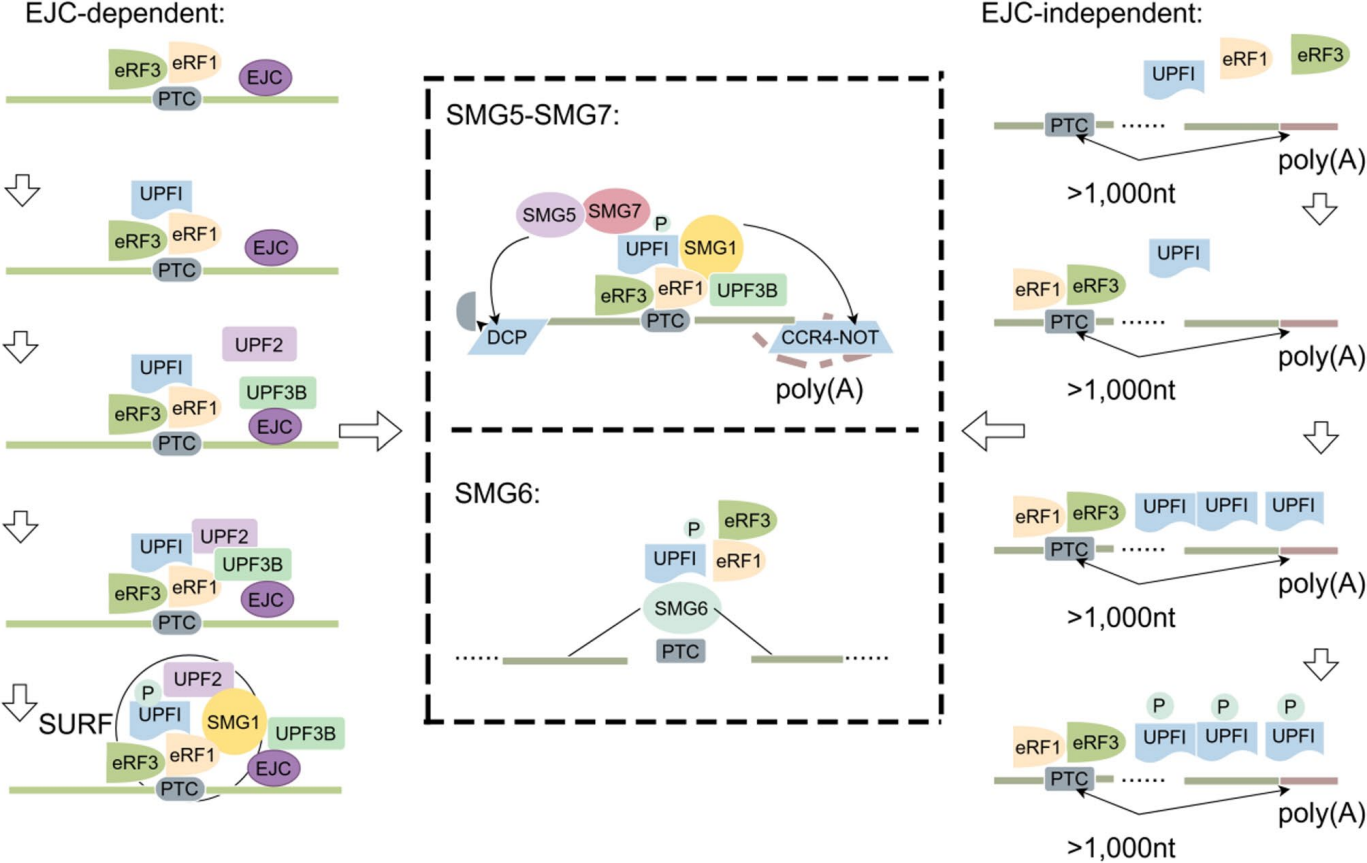
Dual nonsense-mediated mRNA decay (NMD) activation routes. In the classical EJC-dependent pathway (left), UPF3 binds to exon junction complexes (EJCs) downstream of a premature termination codon (PTC), subsequently recruiting UPF2, and activating UPF1 phosphorylation via SMG1. In the EJC-independent pathway (right), transcripts with long 3′ untranslated regions (3’UTRs, typically > 1000 nt) impair the recruitment of eRF1 and eRF3, thereby increasing UPF1 phosphorylation. Phosphorylated UPF1 promotes the degradation of the aberrant transcript via two mechanisms: (1) recruiting the SMG5-SMG7 complex, which promotes mRNA decapping via DCP and deadenylating via the CCR4-NOT complex; (2) recruiting SMG6, an endonuclease that cleaves aberrant mRNAs near the PTC

Thus, NMD operates through multiple routes. Although the classical pathway depends on EJC deposition downstream of a PTC, the EJC-independent route expands the scope of NMD, allowing cells to target aberrant mRNAs that lack splicing-derived junction markers (Fig. 1).

### Biologic rhythm of NMD execution

#### NMD substrates

**Fig. 2:**
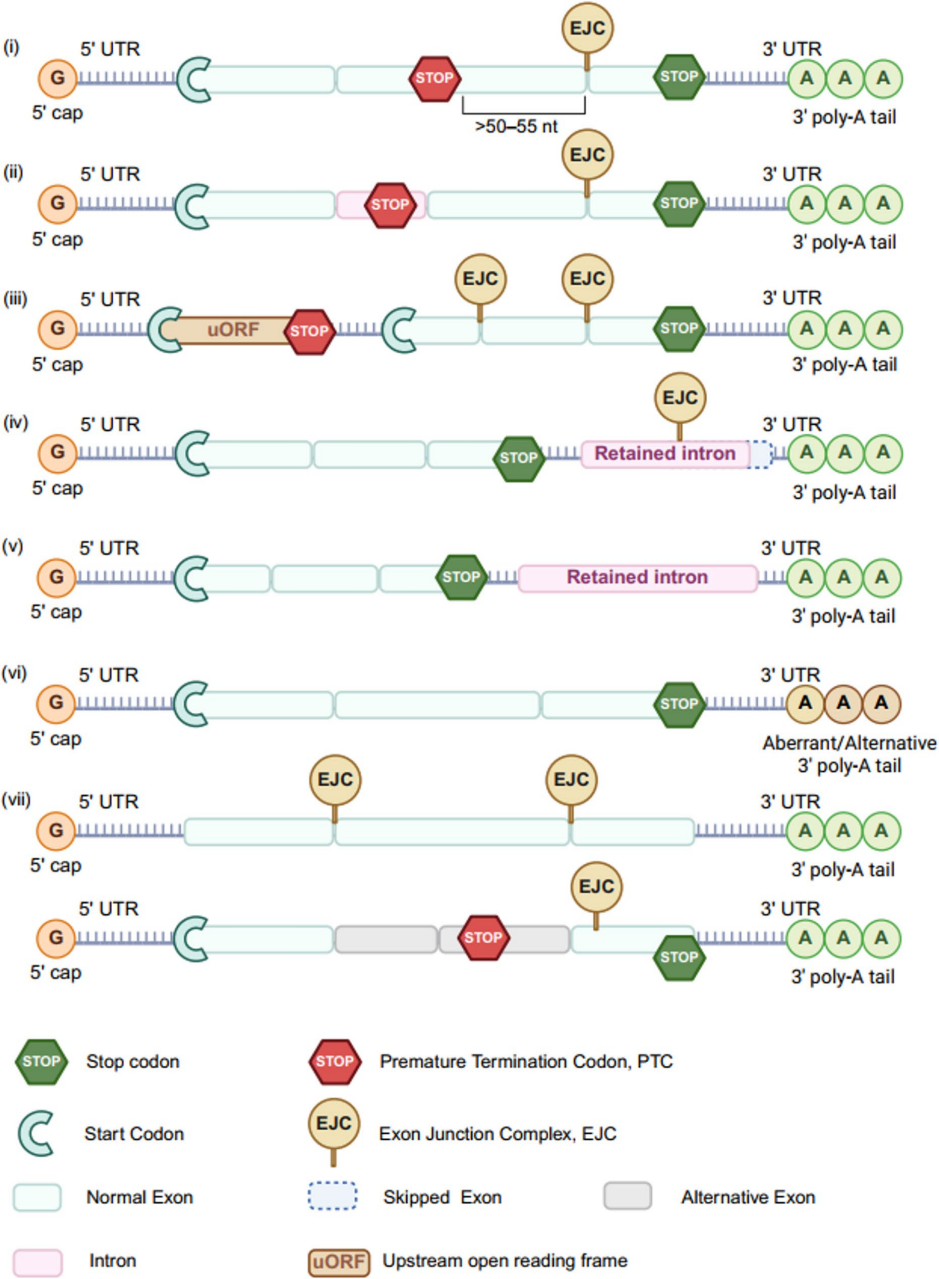
Representative substrates of nonsense-mediated mRNA decay (NMD). (i) mRNAs containing premature termination codons (PTCs) located more than 50–55 nucleotides upstream of exon–exon junctions, are efficiently recognized by the NMD machinery. (ii) PTCs residing within introns in coding regions can also activate NMD. (iii) Transcripts with upstream open reading frames (uORFs) are common NMD targets. (iv) Introns retained in the 3’UTR can preserve the EJCs after translation termination, and induce NMD. (v) Retained introns within the 3’UTR serve as the NMD signals. (vi) Aberrant poly(A) site usage or alternative polyadenylation generates NMD-sensitive transcripts. (vii) Selectively spliced pre-mRNAs and long noncoding RNAs (lncRNAs) are also targeted by NMD

NMD targets a broad spectrum of RNA transcripts with diverse structural features (Fig. 2). Major classical NMD targets include mRNAs harboring PTCs within internal exons. In these cases, the location of the EJCs relative to the exon–exon junction is critical [21]. Transcripts with a PTC occurring upstream of the junction are typically recognized and degraded by the NMD machinery [46, 47]. In mammals, this generally corresponds to PTCs located more than 50–55 nucleotides upstream of the final exon–exon junction [1, 47].

Another classical NMD substrate includes mRNA where the stop codon resides at the end of the exon. Two models have been proposed to explain how these transcripts are recognized. One emphasizes the physical distance between the stop codon and cytoplasmic polyadenylate-binding protein-1 (PABPC1). When this distance increases, the interaction between eRF3 andPABPC1 weakens, promoting NMD activation [48, 49]. The other model, known as the faux-3’UTR model, suggests that a failed interaction mimics premature termination even in the absence of canonical PTCs [34, 50].

Upstream open reading frames (uORFs) represent another important class of NMD targets [17, 35]. These short coding regions, located in the 5’UTR, initiate translation independently and often terminate prematurely, leading to NMD activation [35, 51]. For example, a 44-codon uORF in ZNRD1–AS1 activates NMD and promotes degradation of the full-length transcript, thereby regulating glioma angiogenesis through miR-499a-5p signaling [52]. Similarly, elongation of a uORF in the thrombopoietin gene from 27 to 40 codons renders the transcript NMD-sensitive [53].

The structure and composition of the 3’UTR also influence NMD sensitivity. Although long 3’UTRs are often associated with NMD, some mRNAs with extended 3’UTRs evade degradation through specialized structural or sequence features (as discussed in the section on EJC-independent NMD [37, 38]),. In general, the interplay between 3’UTR configuration, splicing patterns, and associated RNA-binding proteins determines whether a transcript becomes NMD-sensitive.

Additional features can also render transcripts susceptible to NMD. For instance, alternative polyadenylation or mutations in poly(A) signals can impair termination efficiency and disrupt the interaction between the MLLE domain of PABPC1 (residues 544 to 626) and the N-terminal PAM2 motif-containing region of eRF3, promoting decay [54, 55]. Moreover, retained introns, selectively spliced isoforms and certain long noncoding RNAs (lncRNAs) can serve as NMD targets [33, 56, 57].

In summary, NMD recognizes a wide range of transcript features, including PTCs, structured or misprocessed 3’UTRs, uORFs, defective poly(A) usage, and intron retention [39, 58]. This diversity underscores the complexity and adaptability of the NMD surveillance system (Table 1).

**Table 1:** Substrates involved in NMD and example targets for different NMD-inducing substrates

Substrates	Example gene	Related disease/context	Functional Consequence
Upstream ORFs (uORFs)[53]	TPO (Thrombopoietin)	Hepatoma/platelet regulation	Inhibits translation; modulates NMD
Premature termination codons (PTCs)[59, 60]	TP53, DMD (Dystrophin)	Cancer, Duchenne muscular dystrophy	Triggers NMD; reduces protein expression
Long 3′-UTRs[61]	AR (Androgen receptor); CAMK2A (Ca2+/calmodulin-dependent protein kinase II alpha)	Neuronal inflammation, prostate cancer	Activates NMD; lowers mRNA stability
Intronic or alternative polyadenylation (IPA)[62]	DICER1 (Dicer 1, ribonuclease III), FOXN3 (Forkhead Box N3)	Chronic lymphocytic leukemia	Produces NMD-evading truncated mRNAs
lncRNA-derived transcripts[63]	SNHG5 (Small nucleolar RNA host gene 5)	Glioblastoma multiforme	Interacts with UPF1; alters NMD efficiency
AS‑NMD coupled transcripts[64]	SRSF3 (Serine/arginine rich splicing factor 3)	Various cancers	Splicing yields NMD-targeted isoforms

#### Efficiency of NMD

The efficiency of NMD is influenced by both cis-acting mRNA features and trans-acting cellular factors [65]. Although the specific sequence of the PTC itself does not influence NMD strength, several features downstream of the PTC are critical. For example, the nucleotide sequence of the downstream exon can modulate the cleavage rate of NMD [66]. The presence of multiple downstream introns tends to increase NMD efficiency, likely by increasing the number of EJCs that participate in target recognition [33, 66].

The distance between the PTC and the nearest downstream intron strongly affects degradation efficiency [66–68]. Longer distances decrease the physical proximity between the stalled ribosome and the EJC-associated UPF1, compromising the recruitment of decay-promoting complexes. Conversely, very short PTC-intron distances may also impair NMD, possibly because of incomplete ribosome displacement of nearby EJCs, resulting in inefficient surveillance [10, 39].

The position of the PTC relative to the start codon is another important determinant. PTCs located within 200 nucleotides of the start codon result in only 35% NMD efficiency, whereas more distal PTCs exhibit an efficiency of up to 93% [65, 69]. Nevertheless, residual NMD activity can still be observed for proximal PTCs, presumably due to the partial retention of EJCs too close to the stop site [66].

In addition to mRNA-intrinsic features, trans-acting regulatory proteins also modulate NMD efficiency. For example, in plant models, DEAD-box RNA helicases homologous to DDX3 and DDX6 have been shown to increase or inhibit NMD, respectively. These effects possibly arise from helicase-mediated remodeling of RNA structures or altered assembly of NMD complexes.

The complex interplay between transcript structure, translation dynamics, EJC positioning, and auxiliary regulatory proteins shapes the transcript-specific sensitivity to NMD. This multifactorial regulation influences gene expression outcomes in both physiological and pathological contexts.

#### NMD escape

Although NMD targets an extensive range of transcripts, certain RNAs escape degradation even when they possess canonical NMD-inducing features [58, 70]. These NMD escape events may serve important regulatory purposes and are particularly relevant in developmental programs, tissue-specific expression, and stress response.

One major mechanism of NMD escape involves cis-acting inhibitory elements within the mRNA, particularly in the 3’UTR [38, 71]. Some long 3’UTR transcripts contain structured RNA elements or specific sequence motifs within the first 200 nucleotides downstream of the stop codon. These motifs prevent efficient UPF1 recruitment or mimic proper translation termination signals, and prevent degradation [38].

Alterations in translation termination dynamics can also promote NMD evasion. When termination occurs efficiently near PABPC1, its interaction with eRF3 stabilizes the transcript and suppresses NMD [54]. Notably, PABPC1 binding within approximately 35 nucleotides downstream of a PTC strongly inhibits NMD [49, 72]. Similar effects have been observed in *Drosophila* cells, in which PABPC1 blocks NMD activation [49]. In addition to PABPC1, other RNA-binding proteins (RBPs) can also stabilize transcripts by interacting with core NMD factors and preventing their activation [73, 74].

In some cases, alternative splicing events can also lead to NMD escape. Inefficiently spliced introns downstream of the PTC can generate isoforms that may lack EJCs at critical positions, thereby preventing EJC-dependent NMD activation [12]. Transcripts can also resist decay through translational read-through and reinitiation at the PTC, which removes the downstream EJC [75–77]. This mechanism is frequently used by genes involved in immune regulation and developmental signaling, allowing the conditional expression of isoforms that would otherwise be sensitive to NMD.

Cellular stress, such as hypoxia, viral infection, and endoplasmic reticulum (ER) stress, can induce global NMD suppression [78, 79]. This suppression is often mediated by phosphorylation of the translation initiation factor eIF2α, which limits translation reinitiation and alters ribosome occupancy, indirectly reducing NMD efficiency [79–81]. As a result, transcripts that would normally be degraded can accumulate and exert regulatory effects during stress adaptation [82, 83].

Moreover, tissue-specific expression of NMD factors can lead to physiological NMD escape. Low UPF3B or SMG1 levels in certain cell types are associated with impaired NMD activity, allowing transcripts with PTCs or extended 3’UTRs to evade degradation [84–86]. In contrast, SMG1 induction increases NMD in tumors and suppresses frameshift-neoantigen expression [87].

Overall, NMD escape is achieved through a combination of transcript-intrinsic features, dynamic translation termination, alternative splicing, and context-dependent modulation of NMD factor availability. Among these, the ability of a transcript to facilitate translational restart following premature termination has emerged as the key determinant of NMD resistance [75, 88]. Interestingly, NMD efficiency remains reduced even when the downstream AUG lies in a different reading frame, suggesting the existence of additional, uncharacterized mechanisms underlying NMD evasion. Further exploration is needed to fully elucidate these regulatory layers.

### NMD and cancer progression

**Fig. 3:**
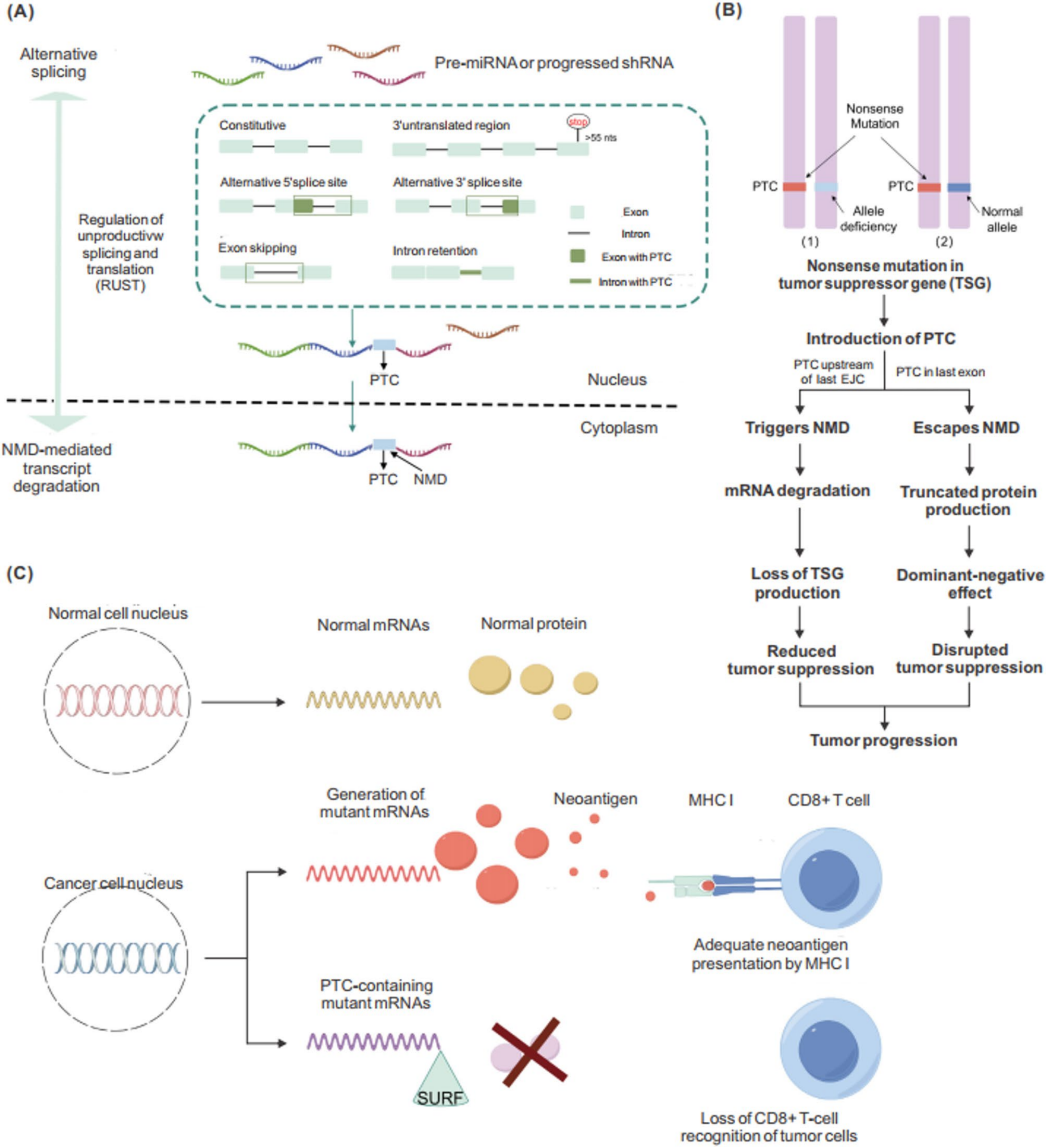
Crucial role of NMD in cancer. **A** Alternative splicing can generate premature termination codons (PTCs), triggering NMD that either promotes or suppresses tumorigenesis. **B** Nonsense mutations in tumor suppressor genes (TSGs) may trigger or escape NMD, leading to tumorigenesis. **C** NMD limits the expression of abnormal peptides and defective proteins, helping cancer cells evade the immune system

Recent studies have established NMD as a key player in tumorigenesis and cancer progression [69, 89]. This section explores the multifaceted interplay between NMD and cancer biology, especially on how oncogenic mutations disrupt gene expression patterns via NMD-associated mechanisms (Fig. 3).

#### Crosstalk between alternative splicing and NMD in cancer

Alternative splicing (AS) is a key regulatory mechanism that increases transcriptomic and proteomic diversity. It is frequently observed during development and lineage-specific differentiation [90, 91]. By selectively including or excluding exons, AS allows cells with identical genomes to generate distinct protein isoforms [92, 93]. When coupled with NMD, AS becomes a powerful post transcriptional system that shapes gene expression and maintains cellular homeostasis. This synergistic AS-NMD mechanism not only regulates physiological processes but also plays dual roles in tumor development [69].

Mechanistically, AS occurs in the nucleus following transcription where precursor mRNAs undergo exon and intron removal and ligation, leading to the generation of multiple transcript isoforms with potentially diverse coding capacities [66]. Although AS increases proteome complexity, this process can also introduce PTC through exon skipping, intron retention, or frameshifts caused by selective splicing site usage [94, 95]. These abnormal transcripts are primed for NMD substrates, which are involved in the recognition and degradation of abnormal mRNAs [92, 96]. Approximately one-third of AS events are estimated to produce transcripts targeted by NMD. As illustrated in Fig. 3A, precursor mRNAs that acquire PTCs during splicing and recombination are subsequently degraded in the cytoplasm, and this activity can either suppress or promote tumor development depending on context.

The coordinated action of AS and NMD is known as regulated unproductive splicing and translation (RUST). In this regulatory system, splicing factors modulate their own expression by producing PTC-generating splice isoforms that are degraded by NMD [91, 95]. RUST helps maintain protein homeostasis by fine-tuning transcript levels and preventing the accumulation of dysfunctional proteins [95, 97].

Dysregulation of AS-NMD contributes to cancer by altering the expression of tumor-related genes [98–102]. For example, under normoxia, AS-NMD limits the expression of cysteine-rich angiogenic inducer 61 (CYR61) by promoting the degradation of specific splice isoforms. However, under hypoxia, tumor cells may reprogram splicing patterns to bypass NMD and increase CYR61 protein levels, thereby increasing survival and promoting tumor progression [103].

Similarly, mutations in splicing regulators such as serine/arginine-rich splicing factor 2 (SRSF2) drive hematological malignancies, including myelodysplastic syndrome (MDS) [104, 105]. SRSF2 mutations alter RNA binding specificity and disrupts splicing fidelity. SRSF2 mutation led to the inclusion of a PTC-bearing exon in the enhancer of the zeste homolog 2 (EZH2) gene, producing an aberrant transcript that is degraded via AS-NMD. This process ultimately restricts EZH2 protein levels and affects tumor growth and development [106, 107].

AS-NMD also has oncogenic potential. Splicing regulators such as SRSF1 and SRSF3 promote the production of oncogenic isoforms of the Ron gene (also known as MST1R, macrophage-stimulating 1 receptor), which increase migration, invasion, and resistance to apoptosis [108, 109]. In these cases, AS-NMD may act as a protective mechanism. For instance, SRSF1-induced splice variants containing PTCs are eliminated by NMD, reducing their tumor-promoting potential. Similarly, SRSF3 overexpression in cancers is counterbalanced by AS-NMD-mediated degradation of its own unproductive mRNA isoforms, thereby limiting its oncogenic effects [110–112].

Through these regulatory loops, AS-NMD contributes to both tumor suppression and progression, depending on the cellular context and mutational background [69, 113]. This multifaceted mechanism highlights the importance of AS-NMD as a critical node in post-transcriptional gene regulation in cancer biology.

#### NMD-mediated regulation of oncogenes and tumor suppressor genes

NMD can contributes to tumorigenesis through both inhibition and activation, depending on the cellular context. Tumors often exploit NMD to degrade transcripts of tumor suppressor genes (TSGs) harboring PTCs, thereby reducing their tumor-suppressive capacity [89, 114]. For instance, E-cadherin (CDH1) transcripts containing PTCs are degraded by NMD, resulting in reduced expression in gastric tissues and promoting hereditary diffuse gastric cancer [115]. Similarly, nonsense mutations in breast cancer gene 2 (BRCA2) activate NMD, elevating the risk of breast and ovarian cancer [116].

Hypermethylated CpG dinucleotides in TSGs are prone to spontaneous deamination of 5-methylcytosine (5mC) or to replication errors, resulting in C-to-T transitions that generate PTCs [6, 117]. These mutations often trigger NMD, further suppressing TSG expression. Tumors also frequently combine PTC-bearing alleles with heterozygous deletions, leading to biallelic inactivation [118]. Even without complete gene loss, the degradation of one single allele can result in haploinsufficiency, which can promote tumor formation [119, 120]. Alternatively, the TSG transcript may evade NMD because of its structural features, allowing the production of truncated proteins that are either partially functional or act as dominant-negative factors [121, 122].

Taken together, tumors may inactivate TSGs via three primary mechanisms involving NMD: (1) combining PTC-bearing alleles with heterozygous deletions, (2) exacerbating haploinsufficiency via degradation of mutant transcripts, and (3) generating dominant-negative proteins from NMD-insensitive transcripts [118–122]. These mechanisms are conceptually illustrated in Fig. 3B.

Although much attention has focused on TSGs, the interaction between oncogenes and NMD is also significant. Oncogenes tend to accumulate missense rather than nonsense mutations [6], perhaps reflecting selective pressure to retain partial function. This pattern suggests that the targeting of oncogenes by NMD warrants may warrant further exploration.

In addition to mutated genes, NMD modulates the expression of normal transcripts that support tumor progression, including mRNAs encoding stress-response factors, signaling proteins, and noncoding RNAs that support tumorigenic phenotypes. In such cases, enhancing NMD potentially suppresses tumor growth, whereas its inhibition exacerbates malignancy [123].

Taken together, these findings suggest that NMD functions as a double-edged sword in cancer biology. Targeting NMD to restore TSG expression or suppress oncogenic transcripts may provide a promising strategy for mRNA-based anticancer therapies.

#### Role of NMD in tumor immune evasion and immunogenicity

Immune surveillance enables the immune system to detect or remove abnormal or malignant cells. A critical component of this defense mechanism is the recognition of tumor-specific-antigens. Frameshift insertion and deletion (indels), which are common in tumor cells, introduce novel open reading frames that produce aberrant proteins, known as neoantigens [124, 125]. These neoantigens are absent in normal tissues and thus recognized as foreign by the host immune system, triggering an antitumor response [125].

NMD strongly influences this immune surveillance mechanism by limiting neoantigen availability. Tumor-associated mutations often generate mRNAs bearing PTCs, which are degraded by NMD before translation [126]. As a result, the potential for neoantigen presentation is diminished, allowing tumor cells to evade immune surveillance. This diminished antigen load allows tumor cells to evade immune detection, as illustrated in Fig. 3C [124, 126]. This immune escape mechanism supports tumor development and maintenance [127].

Multiple studies have provided experimental evidence supporting the immunomodulatory role of NMD. In cancers such as osteosarcoma [128]and colorectal cancer [129], high NMD activity is correlated with a reduced neoantigen load and diminished immune cell infiltration in the TME [130, 131]. Conversely, ablation of the core NMD factor UPF1 stabilizes aberrant transcripts and enhances immune recognition in multiple cancer models [132, 133].

In addition to directly modulating neoantigen expression, NMD influences tumor immunogenicity through its effects on immune signaling pathways and noncoding RNAs. For example, NMD degrades transcripts that regulate interferon responses or antigen presentation machinery, thereby modulating immune sensitivity to immune attack [134, 135].

Taken together, these findings reveal that NMD shapes the tumor–immune interface by modulating neoantigen availability and influencing immune surveillance. These findings suggest that its modulation potentially represent a promising target for enhancing immunotherapeutic efficacy.

#### NMD-driven modulation of the tumor microenvironment

The tumor microenvironment (TME)consists of immune cells, fibroblasts, vasculature, extracellular matrix components and various signaling molecules. Increasing evidence suggests that NMD interacts with the TME, and influences both tumor progression and immune modulation [89, 126, 136].

Under cellular stress conditions, such as ER stress, NMD activity is often downregulated [81, 82]. This suppression is largely related to the phosphorylation of eIF2, which inhibits translational reinitiation and stabilizes stress-responsive transcripts such as ATF-4, ATF-3, and CHOP. These mediators promote tumor cell adaptation to hypoxia and nutrient deprivation, thus facilitating tumor survival and growth [137, 138]. Recent studies have revealed a reciprocal regulatory relationship between NMD and ER stress signaling. The core NMD factor UPF3B interacts directly with the ER stress sensor IRE1α to inhibit its kinase activity, autophosphorylation, and clustering during stress [137]. This interaction is further modulated by the UPF3B phosphorylation status.

Moreover, tumor-intrinsic signaling increases NMD activity. IL-6/STAT3 signaling in tumor cells induces SMG1 and enhances NMD activity, which limits the expression of effective coding-altered neoantigens and weakens the immune surveillance [87]. Conversely, maintaining UPF1 expression suppresses tumorigenesis by preserving transcriptome stability and NMD activity. In hepatocarcinogenesis, UPF1 degradation facilitates malignant progression [139–141].

Defective NMD also contributes to remodeling of the TME. For example, damaged NMD reduces the expression of the tumor suppressor CYLD, a deubiquitinase that negatively regulates the NF-κB pathway [142, 143]. The loss of CYLD sustains NF-κB signaling, which promotes chemokine production and immune cell infiltration, as observed in inflammatory myofibroblasts [142, 144, 145].

### Clinical implications and translational potential of NMD

**Fig. 4:**
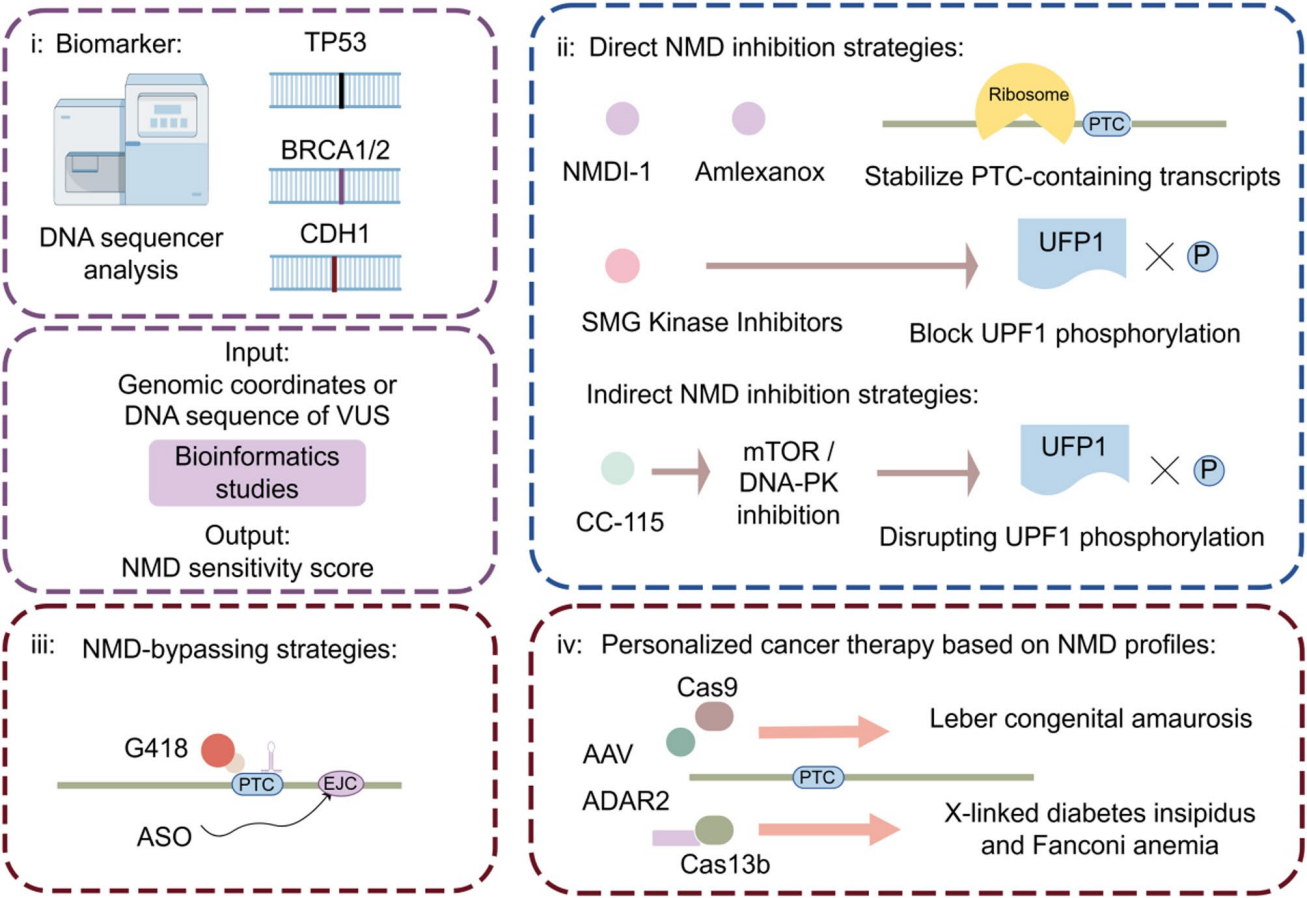
Diagnostic and therapeutic strategies involving NMD. (i) NMD as a diagnostic and prognostic biomarker. (ii) Direct NMD inhibition strategies include small-molecule inhibitors (e.g., MDI-1/Amlexanox), kinase inhibitors (e.g., CC-115), siRNA knockdown of core NMD factors (e.g., UPF1, SMG7), and antisense oligonucleotides (ASOs). (iii) NMD-bypassing strategies: Readthrough-inducing antibiotics (e.g., G418) or ASOs bypass NMD activation by modifying transcript features. (iv) Gene-editing-based correction: CRISPR and RNA-editing tools (e.g., Cas9, Cas13b, ADAR2) enable precise correction of NMD-sensitive mutations in genetic diseases and cancer

Mechanistic insights into NMD have opened new avenues for translational cancer research (Fig. 4). As NMD regulates tumor suppressors, oncogenes, and neoantigens, its modulation holds potential for diagnostic, prognostic, and therapeutic applications. In this section, we explore current clinical translation efforts and their implications for precision oncology [89, 124, 146].

#### NMD as a diagnostic and prognostic biomarker

Given its role in transcript stability, NMD activity can serve as a biomarker for tumor progression and treatment response. Tumors harboring nonsense mutations in key tumor suppressor genes, such as TP53, BRCA1/2, or CDH1, often exhibit increased NMD activity. This finding is correlated with reduced protein expression and more aggressive phenotypes [124, 147–149]. For instance, high NMD efficiency in colorectal or breast cancers with PTC-containing transcripts has been associated with poor clinical outcomes [69, 149, 150].

RNA-seq-based assessments of NMD activity, inferred from transcript isoform ratios or NMD-sensitive transcript abundance, are currently under investigation for stratifying patients into prognostic subgroups [146, 151].

Recent bioinformatics studies have incorporated NMD efficiency and transcript features into prognostic models. These include parameters such as readthrough potential and isoform-specific expression patterns [152]. Tools such as NMDetective, which predict NMD sensitivity using sequence-based features, aid in the classification of variants of uncertain significance (VUSs) in tumor suppressor genes. This is especially relevant for interpreting variants in BRCA2 or MSH2, where NMD involvement may influence cancer risk and therapeutic decisions [146].

#### Targeting NMD for cancer therapy

Pharmacological suppression of NMD has gained attention as a promising strategy in cancer therapy, particularly for enhancing tumor immunogenicity and restoring the expression of tumor suppressor genes containing PTCs [153].

Several small-molecule inhibitors directly target core NMD components. For example, NMDI-1 [154] and amlexanox [155] suppress NMD activity, resulting in the stabilization of PTC-containing transcripts and facilitating the re-expression of truncated tumor suppressor proteins or neoantigens. SMG1 and SMG7 kinase inhibitors are another class of agents explored for NMD inhibition, as SMG-mediated UPF1 phosphorylation is a crucial step in NMD activation [156, 157]. Compounds such as CC-115, a dual mTOR and DNA-PK inhibitor, indirectly suppress NMD by interfering with upstream kinases responsible for UPF1 phosphorylation, and have shown promise in the treatment of multiple myeloma [156]. However, achieving high specificity remains a major challenge because SMG1 and related kinases also participate in other essential cellular pathways, including the DNA damage response [158, 159].

In addition to small molecules, genetic approaches have been investigated. Antisense oligonucleotides (ASOs) or siRNA-mediated knockdown of core NMD factors such as UPF1 or SMG7 selectively impaired NMD in preclinical studies and restored the expression of genes suppressed through nonsense mutations [126, 157, 160].

NMD inhibition has also been shown to synergize with immunotherapies. NMD inhibition reveals silent neoantigens by allowing the expression of mutated proteins that would typically be degraded. This is particularly relevant in tumors with high mutational burden, such as microsatellite instability-high (MSI-H) cancers, where frameshift mutations frequently generate PTC-containing transcripts. Inhibiting NMD in these tumors can increase neoantigen presentation, improve T-cell recognition and strengthen antitumor immunity [124]. For example, UPF1 knockdown enhances antigen presentation and sensitizes colorectal cancer cells to immune checkpoint blockade with anti–PD-1 antibodies [133]. Such findings support that transient and localized NMD suppression could be integrated into immunotherapy regimens, particularly for immunologically “cold” tumors.

Despite its potential, systemic NMD inhibition may stabilize aberrant transcripts in normal cells. This risk indicates the need for precise delivery and selective targeting of NMD-modulating agents [161–163].

#### Exploiting NMD bypass strategies

Rather than directly inhibiting the NMD machinery, an alternative approach is to bypass NMD activation by modifying transcript features, thereby preventing recognition and degradation. These strategies include PTC readthrough, exon skipping, and the modulation of EJC deposition or translation termination [164–168].

PTC readthrough can be induced by aminoglycoside antibiotics (e.g., G418 and gentamicin) [169–171] and nonaminoglycoside molecules (e.g., Ataluren/PTC124) [168, 172–174]. These compounds promote ribosomal misreading of premature stop codons, allowing translation to proceed and full-length or near-full-length proteins to be produced. It was initially developed for genetic diseases (e.g., cystic fibrosis and Duchenne muscular dystrophy), and has since been repurposed for cancer models [172, 175]. For instance, Ataluren facilitates PTC readthrough by modulating release factor-dependent termination and has shown efficacy in preclinical models of PTC-driven cancers [168]. Combining G418 with CDX5-1 significantly increased PTC readthrough in cells harboring nonsense mutations in TP53, CLN2, SMARCAL1 and DMD [176]. However, the therapeutic index remains narrow because of toxicity and unintended off-target readthrough of natural stop codons [171, 177].

ASOs could offer versatile strategies to bypass NMD [165, 178, 179]. One approach involves designing ASOs to induce exon skipping of PTC-containing exons, and removing nonsense mutations from the mature transcript [179–181]. Although the resulting proteins may lack certain domains, they can retain partial function and offer a therapeutic alternative when full gene replacement is impractical. Another strategy involves the use of ASOs to disrupt EJC assembly near PTCs, preventing aberrant recognition and allowing transcripts to escape degradation [165, 182].

Collectively, these bypass strategies minimize interference with overall NMD activity, focusing instead on selectively rescuing transcripts of therapeutic interest. Although most data remain preclinical, the continued optimization of ASO chemistry and PTC-readthrough agents holds promise for clinical translation [152, 170, 183].

#### Personalized cancer therapy based on NMD profiles

The heterogeneity of NMD activity and mutational burden among patients provides a foundation for personalized cancer therapies. By profiling tumor-specific PTCs and NMD efficiency, treatment strategies can be tailored to restore the expression of key transcripts otherwise suppressed by NMD.

Recent advances in clustered regularly interspaced short palindromic repeats (CRISPR)-based editing have enabled targeted correction of nonsense mutations [184, 185]. In a clinical trial for Leber congenital amaurosis (LCA10), adeno-associated virus (AAV) delivery of Cas9 nucleases and guide RNAs successfully targeted the underlying mutation and achieved functional improvements in vivo [186, 187]. Such approaches hold translational potential for cancers harboring NMD-sensitive tumor suppressor mutations.

Base editors further expand this toolbox by coupling catalytically inactive Cas enzymes with nucleotide-modifying domains, enabling precise correction without DNA breaks [188]. For instance, the CRISPR-PASS system corrected a nonsense mutation in the XPC gene, and achieved protein restoration in patient-derived cells [189]. RNA-targeted editing also offers transient alternatives [190]. The fusion of ADAR2 and inactive Cas13b has been used to edit PTCs in diseases such as X-linked diabetes insipidus and Fanconi anemia, providing a flexible strategy without permanent genomic modification [190, 191].

Together, these technologies suggest a future in which NMD profiling guides individualized cancer therapy, especially in tumors rich in PTCs or with high mutational burdens. Through readthrough induction, CRISPR correction, or RNA editing, restoring the expression of tumor suppressors or neoantigens may significantly improve clinical outcomes.

## Discussions

Traditionally, NMD has been regarded as a quality control mechanism that eliminates mRNAs harboring PTCs. By preventing the production of truncated, potentially deleterious proteins, NMD has long been thought to safeguard genomic integrity and maintain cellular homeostasis. However, accumulating evidence has revealed that this is overly simplistic. In oncology, NMD plays a dual role: although it can suppress tumorigenesis by eliminating mutated or nonfunctional mRNAs, it can also contribute to tumor progression by degrading TSG transcripts and neoantigen-coding mRNAs [69, 124].

Over the past two decades, insights derived from genetic, transcriptomic, and preclinical models have substantially improved our understanding of the role of NMD in cancer biology. These studies revealed that whether NMD affects oncogenic or tumor-suppressive outcomes depends heavily on the mutational context and tumor type [89, 157]. Furthermore, the intricate crosstalk between AS and NMD adds another layer of complexity to gene expression regulation. This AS-NMD interplay allows cells to fine-tune transcript levels dynamically and can promote or inhibit tumorigenesis depending on the cellular content [91, 92, 107].

Therapeutically, strategies targeting or bypassing the NMD pathway have emerged as promising approaches for cancer treatment. Direct NMD inhibition using small-molecule inhibitors (e.g., NMDI-1 [76], CC-115 [156]) or genetic suppression of NMD factors (e.g., UPF1 knockdown [133]) can restore the expression of TSGs or enhance tumor antigenicity. In parallel, NMD bypass strategies, including PTC readthrough inducers such as Ataluren [168, 192] or exon-skipping antisense oligonucleotides [193], enable the restoration of protein expression without completely disabling the NMD pathway. Such strategies are especially pertinent in high-mutational burden tumors, where NMD suppresses the expression of frameshift-derived neoantigens [194].

Notably, combining NMD inhibition with immune checkpoint blockade has demonstrated synergistic effects in preclinical models, converting immunologically “cold” tumors into “hot” ones [133, 194]. Advances in RNA-editing technologies, CRISPR-based correction of nonsense mutations, and antisense oligonucleotide design are accelerating this transition toward individualized RNA-based interventions.

Despite these advances, several challenges persist. Systemic NMD inhibition is associated with the stabilization of aberrant transcripts in normal tissues, raising concerns about off-target toxicity [162, 195–197]. Tumor heterogeneity and variable NMD efficiency between individuals complicate the identification of predictive biomarkers and patient stratification. Moreover, many pharmacological and genetic tools remain at the preclinical stage, with limited data on long-term safety, delivery efficiency, and specificity.

**Fig. 5:**
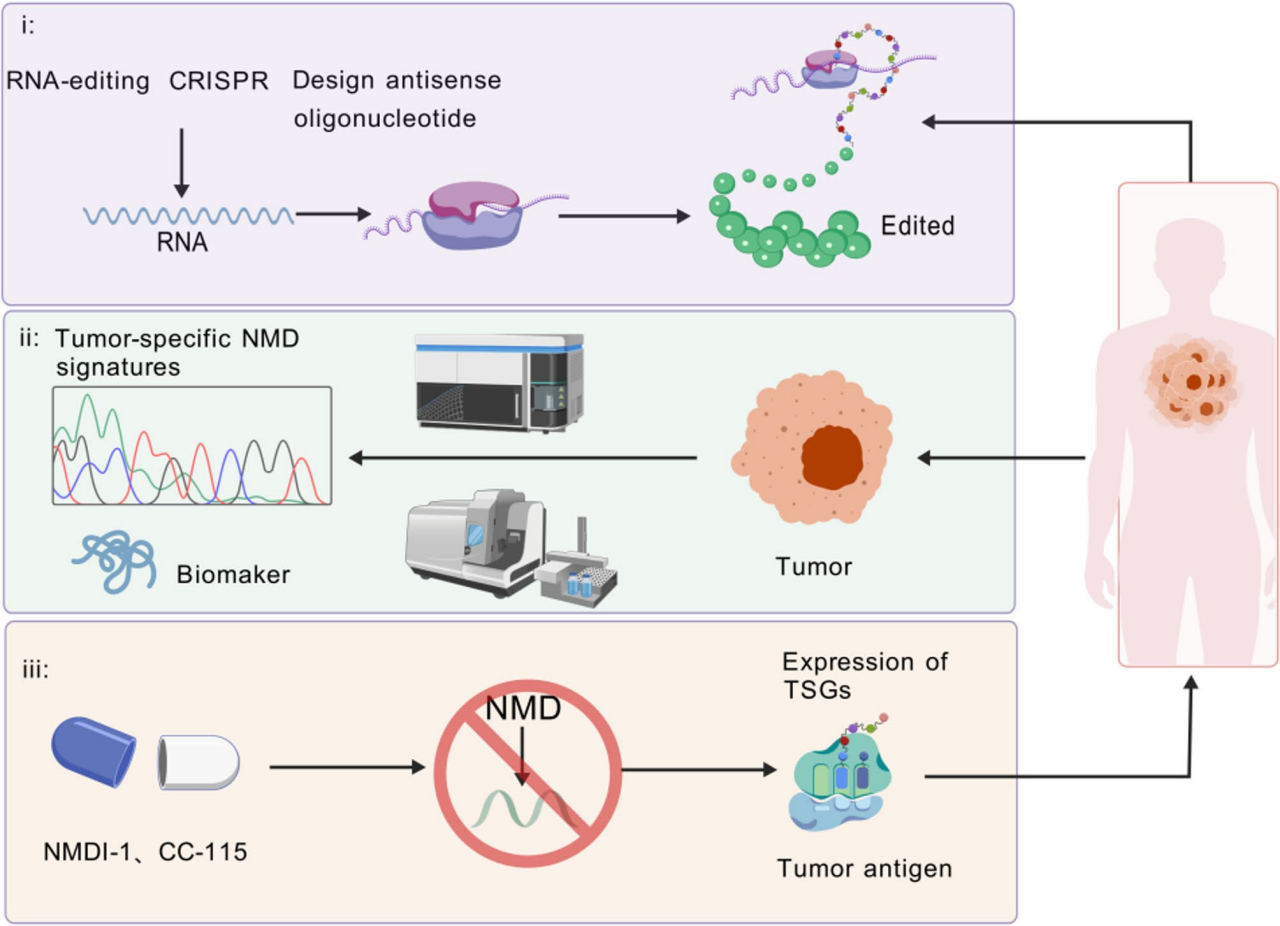
Therapeutic targeting of NMD treatment: current strategies and future prospects. (i) Individualized RNA-based approaches including RNA-editing, CRISPR, and antisense oligonucleotides (ASOs) aim to restore a full-length protein expression from transcripts harboring nonsense mutations. (ii) Tumor-specific NMD signatures guide precise patient stratification and support the development of NMD-targeted therapies. (iii) NMD inhibition by small-molecule inhibitors (e.g., NMDI-1, CC-115) could promote the re-expression of tumor suppressor genes (TSGs) and tumor-associated antigens, providing a potential avenue for precision oncology

Future directions should focus on mapping tumor-specific NMD signatures and integrating them into multiomics frameworks. Elucidating context-dependent regulators of NMD and identifying reliable biomarkers will be essential for optimizing therapeutic efficacy. Ultimately, combining NMD modulation with established therapies, such as immunotherapy or targeted agents, may unlock new opportunities for precision oncology (Fig. 5).

## Conclusions

NMD plays a dual role in cancer, functioning both as a tumor suppressor and as a promoter of tumor progression by eliminating beneficial transcripts. With increasing insights into its interaction with oncogenic signaling, immune evasion, and RNA regulation, The efficacy of NMD has been demonstrated in preclinical studies. To achieve clinical translation, future research must address tumor-specific NMD dependencies, and biomarker development, and evaluate clinical safety to translate these approaches into precision oncology.

## Data Availability

No datasets were generated or analysed during the current study.

## Abbreviations

AAV: Adeno-associated virus
AR: Androgen receptor
AS: Alternative splicing
AS-NMD: Alternative splicing-coupled nonsense-mediated mRNA decay
ASOs: Antisense oligonucleotides
BRCA: Breast cancer gene 2
CAMK2A: Ca²⁺/calmodulin-dependent protein kinase II alpha
CBP: Cap-binding protein
CCR4–NOT: Carbon catabolite repression 4–negative on TATA complex (major eukaryotic deadenylation complex)
CYR61: Cysteine-rich angiogenic inducer 611
DCP: Decapping complex
DICER1: Dicer 1, ribonuclease III
DMD: Dystrophin
EJC: Exon junction complex
eRF1: Eukaryotic release factors 1
eRF3: Eukaryotic release factors 3
ER: Endoplasmic reticulum
EZH2: Zeste homolog 2
FOXN3: Forkhead box N3
indels: Insertion and deletion
IPA: Intronic polyadenylation
LCA10: Leber congenital amaurosis
lncRRNAs: Long non-coding RNAs
MDS: Myelodysplastic syndromes
MSI-H: Microsatellite instability-high
NMD: Nonsense-mediated mRNA decay
PABPC1: Polyadenylate-binding protein-1
PTC: Premature termination codon
RBPs: RNA-binding proteins
RUST: Regulated unproductive splicing and translation
SMG1: Suppressor of morphogenesis in genitalia 1
SNHG5: Small nucleolar RNA host gene 5
SRSF2: Serine/arginine-rich splicing factor 2
SRSF3: Serine/arginine-rich splicing factor 3
SURF: SMG1–UPF1–eRF1–eRF3 complex
TME: Tumor microenvironment
TPI: Triosephosphate isomerase
TSGs: Tumor suppressor genes
uORFs: Upstream open reading frames
UPF1: Up-frameshift protein 1
UPF2: Up-frameshift protein 2
UPF3B: Up-frameshift 3 homolog B
VUS: Variants of uncertain significance
3'UTRs: 3'-untranslated regions
5mC: 5-methylcytosine
